# Development and Implementation of a Hands-on Surgical Pipeline Program for Low-Income High School Students

**DOI:** 10.1001/jamanetworkopen.2019.9991

**Published:** 2019-08-23

**Authors:** Serena S. Bidwell, Miquell O. Miller, Edmund W. Lee, Kirbi Yelorda, Sonia Koshy, Mary Hawn, Arden M. Morris

**Affiliations:** 1 S-SPIRE Center, Department of Surgery, Stanford University, Stanford, California; 2 The Kapor Center, Oakland, California

## Abstract

This qualitative study describes the development and implementation of a hands-on surgical pipeline program for low-income high school students.

## Introduction

Students belonging to racial/ethnic minority groups continue to be underrepresented among medical school applicants, matriculates, and graduates.^1^ While existing pipeline programs for students who are underrepresented in medicine engage them in biomedicine at the undergraduate level, efforts to promote diversity in the medical workforce may be more effective during the years from kindergarten through 12th grade.^2^ We created a summer pipeline program focused on surgery, invited participation by students from low-income high schools, and queried students’ self-efficacy, interest, and perceived barriers in this convergent mixed-methods study. Our emphasis on surgery was intended to address the lower percentage of physicians from underrepresented groups in medical subspecialties.^3,4,5^

## Methods

After conducting a local needs assessment, we partnered with a residential science, technology, engineering, and math (STEM)–intensive, tuition-free pipeline program called Summer Math and Science Honors (SMASH) Academy. Over 3 consecutive years, the program provides high school students from underrepresented groups with the resources, experiences, and academic support to succeed in STEM fields. We developed 5 interactive workshops (health care professional small-group sessions and large-group panel, a patient panel, vital signs, laparoscopic and robotic surgery skills, and prosected cadavers), led by a combination of medical school faculty, staff, surgery residents, and medical students, that emphasized hands-on, practical learning related to surgery while also exposing the students to a range of other health care career options.

Precurriculum and postcurriculum surveys addressed domains based on existing literature: self-efficacy (belief in one’s ability to succeed in achieving a goal), interest in the health care field, and perceived barriers to a health care career. Mean participant scores for the various domains were calculated from values for the 62 participants who completed both the precurriculum and postcurriculum surveys, which used a Likert scale ranging from 1 (not at all agree) to 5 (strongly agree). The Stanford University institutional review board designated this study exempt from review because participants incurred minimal risk. All participants signed informed consent to complete program evaluation surveys.

Descriptive demographic and survey data were collected electronically. Qualitative responses to open-ended survey questions were coded by 2 of us (S.S.B. and M.O.M.) and discrepancies were discussed to consensus. We used paired *t* tests to compare precurriculum and postcurriculum survey responses and a Kruskal-Wallis test to account for skewed data. Analysis was conducted using R statistical software version 1.0.153 (R Project for Statistical Computing). Two-sided *P* < .05 was considered statistically significant. We integrated quantitative and qualitative findings in this convergent mixed-methods study through a joint display.^6^

## Results

Among 88 students, 44 (50%) were female. Fifty-three (61%) self-identified as Latino, 23 (26%) as African American, and 11 (13%) as other race/ethnicity. Sixty-eight (77%) reported receipt of free lunch at their high schools and 71 (81%) indicated that they were future first-generation college students.

**Figure 1.**
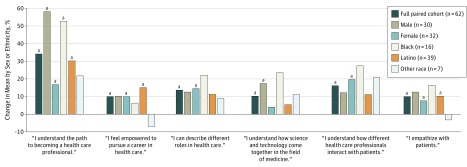
Precurriculum to Postcurriculum Change in Health Care Self-efficacy and Interest by Demographic Group Means were the average values for the 62 participants who completed both the precurriculum and postcurriculum surveys, which used a Likert scale ranging from 1 (not at all agree) to 5 (strongly agree). No significant change was found in the following categories: interest in becoming a clinician, interest in educational opportunities with clinicians, interest in clinical mentors, ability to become a health care professional, excitement about health care career opportunities, and support for pursuing health care. ^a^Denotes statistical significance (*P* < .05).

After the program, students displayed greater self-efficacy (precurriculum mean [SD], 3.37 [1.3] vs postcurriculum mean [SD], 3.71 [1.2]; difference, 0.34; 95% CI, −0.61 to −0.06; *P* = .02) (Figure 1) and endorsed better understanding of the path to a health care career (precurriculum mean [SD], 2.79 [1.3] vs postcurriculum mean [SD], 3.74 [1.0]; difference, 0.95; 95% CI, −1.25 to −0.65; *P* < .001). Students reported improved understanding of the integration of science and technology in the field of medicine (precurriculum mean [SD], 3.65 [1.1] vs postcurriculum mean [SD], 4.02 [0.9]; difference, 0.37; 95% CI, −0.69 to −0.05; *P* = .02). Students also reported a significant increase in knowledge regarding interaction with patients (precurriculum mean [SD], 3.39 [1.1] vs postcurriculum mean [SD], 3.94 [1.0]; difference, 0.55; 95% CI, −0.87 to −0.23; *P* = .001) and improved empathy for patients (precurriculum mean [SD], 3.77 [1.1] vs postcurriculum mean [SD], 4.15 [1.0]; difference, 0.38; 95% CI, −0.64 to −0.10; *P* = .008) (Figure 1).

**Figure 2.**
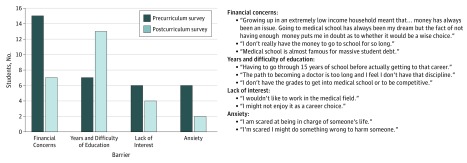
Joint Display of Students’ Perceived Barriers to Pursuit of a Health Care Career

When asked about barriers toward pursuit of a health care career, 35 of 75 students (47%) who completed the precurriculum survey and 42 of 68 students (62%) who completed the postcurriculum survey responded affirmatively. In open-ended responses, students cited concern about a large financial burden, the years of education, and anxiety about or lack of interest in health care careers (Figure 2). A few students mentioned immigrant status, lack of home support, or lack of mentorship in health care as additional barriers.

## Discussion

This mixed-methods study found statistically significant positive changes in self-efficacy and perceived knowledge after exposure to the curriculum. The program aims to continue operating in the coming years, through inclusion of new educational opportunities and strengthening community partnerships. Structured pipeline programs such as this one have the potential to engage high school students from underrepresented groups in hands-on health care activities and to help them envision pursuit of a health care career.
